# Split-Cre-mediated GFP expression as a permanent marker for flagellar fusion of *Trypanosoma brucei* in its tsetse fly host

**DOI:** 10.1128/mbio.03375-24

**Published:** 2024-12-17

**Authors:** Ruth Etzensperger, Mattias Benninger, Berta Pozzi, Ruth Rehmann, Arunasalam Naguleswaran, Gabriela Schumann, Jan Van Den Abbeele, Isabel Roditi

**Affiliations:** 1 Institute of Cell Biology, University of Bern, Bern, Switzerland; 2 Department of Biomedical Sciences, Trypanosoma Unit, Institute of Tropical Medicine, Antwerp, Belgium; University of Geneva, Geneva, Switzerland

**Keywords:** trypanosoma, flagellar fusion, Split-Cre, lineage tracing, tsetse

## Abstract

Trypanosomes have different ways of communicating with each other. While communication via quorum sensing, or by the release and uptake of extracellular vesicles, is widespread in nature, the phenomenon of flagellar fusion has only been observed in *Trypanosoma brucei*. We showed previously that a small proportion of procyclic culture forms (corresponding to insect midgut forms) can fuse their flagella and exchange cytosolic and membrane proteins. This happens reproducibly in cell culture. It was not known, however, if flagellar fusion also occurs in the tsetse fly host, and at what stage of the life cycle. We have developed a split-Cre-Lox system to permanently label trypanosomes that undergo flagellar fusion. Specifically, we engineered trypanosomes to contain a GFP gene flanked by Lox sites in the reverse orientation to the promoter. In addition, the cells expressed inactive halves of the Cre recombinase, either N-terminal Cre residues 1–244 (N-Cre) or C-terminal Cre residues 245–343 (C-Cre). Upon flagellar fusion, these Cre halves were exchanged between trypanosomes, forming functional full Cre and flipping reverse-GFP into its forward orientation. We showed that cells that acquired the second half Cre through flagellar fusion were permanently modified and that the cells and their progeny constitutively expressed GFP. When tsetse flies were co-infected with N-Cre and C-Cre cells, GFP-positive trypanosomes were observed in the midgut and proventriculus 28–34 days post-infection. These results show that flagellar fusion not only happens in culture but also during the natural life cycle of trypanosomes in their tsetse fly host.

**IMPORTANCE:**

We have established a procedure to permanently label pairs of trypanosomes that transiently fuse their flagella and exchange proteins. When this occurs, a reporter gene is permanently flipped from the “off” to the “on” position, resulting in the production of green fluorescent protein. Crucially, green trypanosomes can be detected in tsetse flies co-infected with the two cell lines, proving that flagellar fusion occurs in the host. To our knowledge, we are the first to describe a split-Cre-Lox system for lineage tracing and selection in trypanosomes. In addition to its use in trypanosomes, this system could be adapted for other parasites and in other contexts. For example, it could be used to determine whether flagellar fusion occurs in related parasites such as Leishmania and *Trypanosoma cruzi* or to monitor whether intracellular parasites and their hosts exchange proteins.

## INTRODUCTION

Protozoan parasites employ a variety of different mechanisms to exchange information about their status and their environment (1–3). This can involve the release, uptake, or binding of soluble factors or membrane-bound vesicles, or the formation of intercellular bridges such as nanotubes and the exchange of cytoplasmic contents. In the former case, the information is dispersed and can be detected and interpreted by cells that might be a considerable distance away, whereas the latter is restricted to cells in direct contact and may go unnoticed by other cells in their vicinity.

*Trypanosoma brucei.* subspecies*,* protozoan parasites that causes sleeping sickness in humans and nagana in cattle, are extracellular throughout their life cycle in their mammalian and tsetse fly hosts. While this presents challenges in terms of its exposure to the host immune system, it means that the parasite is always well-placed to sense and transmit information (1–3). In the mammalian host, there are two life-cycle stages, proliferating slender forms and quiescent stumpy forms. The balance between the two stages is regulated by quorum sensing, with peptidases released by slender forms generating oligopeptides that trigger their differentiation to stumpy forms (4, 5). This limits the parasite titer and prolongs the life of the host. Slender forms also extrude flagellar exosomes that can fuse and deliver contents to other trypanosomes or to host cells (6). Procyclic (tsetse midgut) forms of the parasite do not shed exosomes under normal conditions, but the knockdown of spliceosomal proteins leads to a block in spliced leader biogenesis and the release of exosomes containing the spliced leader precursor. These exosomes are perceived as repellents by procyclic forms cultured on semi-solid surfaces (7). Communities of procyclic forms can also sense and migrate together along pH gradients that they generate themselves (8). The response to pH requires cyclic AMP signaling (8, 9). *In vivo*, disrupting signaling by deleting components of the cyclic AMP signaling cascade causes a defect in migration between fly organs (8, 10, 11).

An additional form of cell-cell communication that, to date, has only been reported for trypanosomes, is flagellar fusion and the exchange of membrane and cytoplasmic proteins (12). When procyclic forms labeled with different fluorescent proteins were co-cultured, a small proportion of cells were observed to contain both fluorescent proteins after 24–48 h. Electron microscopy of pairs of double-positive cells revealed that they shared a single flagellum containing two axonemes and two paraflagellar rods surrounded by a single membrane (12). Following flagellar fusion, cells were able to exchange cytoplasmic and membrane proteins bidirectionally. This could occur very rapidly, in less than a minute, provided the proteins could gain access to the flagellum (12). EP procyclin, a GPI-anchored protein that is a major component of the parasite surface coat, and calflagin, a protein attached to the inner membrane, were both exchanged by fused cells. By contrast, NT10, a nucleoside transporter restricted to the cell body, did not translocate to the partner cell (12). It was further shown that flagellar fusion was dependent on extracellular calcium and that its frequency was enhanced in the presence of fresh, non-heat-inactivated fetal bovine serum (FBS). Trypanosomes that lacked the major GPI-anchored surface proteins, procyclins, showed a 10-fold increase in fusion frequency (12). This suggested that other surface molecules were required for fusion and that these had become more accessible. In some cases, flagellar fusion was transient, while in others it persisted for several hours at least, that is, for the duration of the experiment. Cells with fused flagella were able to swim together and even give rise to progeny, suggesting that fusion was not inherently harmful. DNA exchange was not observed, however, nor could double-resistant progeny be isolated after co-culture of cells carrying different selectable markers (12). While these observations are intriguing, it is not understood why a small proportion of the procyclic population undergoes flagellar fusion in culture, what the benefits of flagellar fusion might be, or whether it happens *in vivo* during migration of trypanosomes through their tsetse fly host.

The experimental system previously used to study flagellar fusion *in vitro* is not suitable for *in vivo* studies in tsetse flies. The exchange of fluorescent proteins is transient, and the proteins acquired by fusion become undetectable within 72 h, presumably due to a combination of cell division and protein degradation (12). This would make it hard to identify trypanosomes that had undergone fusion *in vivo*, as any trypanosomes that fused a few days prior to dissection would be missed. To overcome this, we established a system that creates an irreversible change in the genome, allowing lineage tracing of trypanosomes that had undergone flagellar fusion *in vivo*. Cre recombinase, which recognizes and cleaves LoxP sites (13, 14), is used by a bacteriophage to integrate or excise its genome from the host chromosome. This system has been adapted for a range of eukaryotic cells, enabling sequences to be excised or inverted. Here we demonstrate the use of split-Cre and two reporter cassettes as a means of permanently marking cells that have undergone flagellar fusion. Trypanosomes were stably transformed with either the N-terminal (N-Cre) or C-terminal portions of Cre (C-Cre), which are inactive on their own; exchange of the two halves of the recombinase via flagellar fusion transiently creates an active form. In culture, this is sufficient to invert a reverse reporter sequence flanked by synthetic LoxP sites in the trypanosome genome, resulting in the constitutive expression of enhanced green fluorescent protein (GFP). When tsetse flies were co-infected with trypanosomes containing the LoxP-GFP_rev_ reporter cassette and simultaneously expressing N- or C-Cre, green trypanosomes were detected in the fly midgut and in the proventriculus, demonstrating that flagellar fusion had occurred *in vivo*.

## RESULTS

### Generation of Lox-GFP_rev_ cells expressing split-Cre

**Fig 1:**
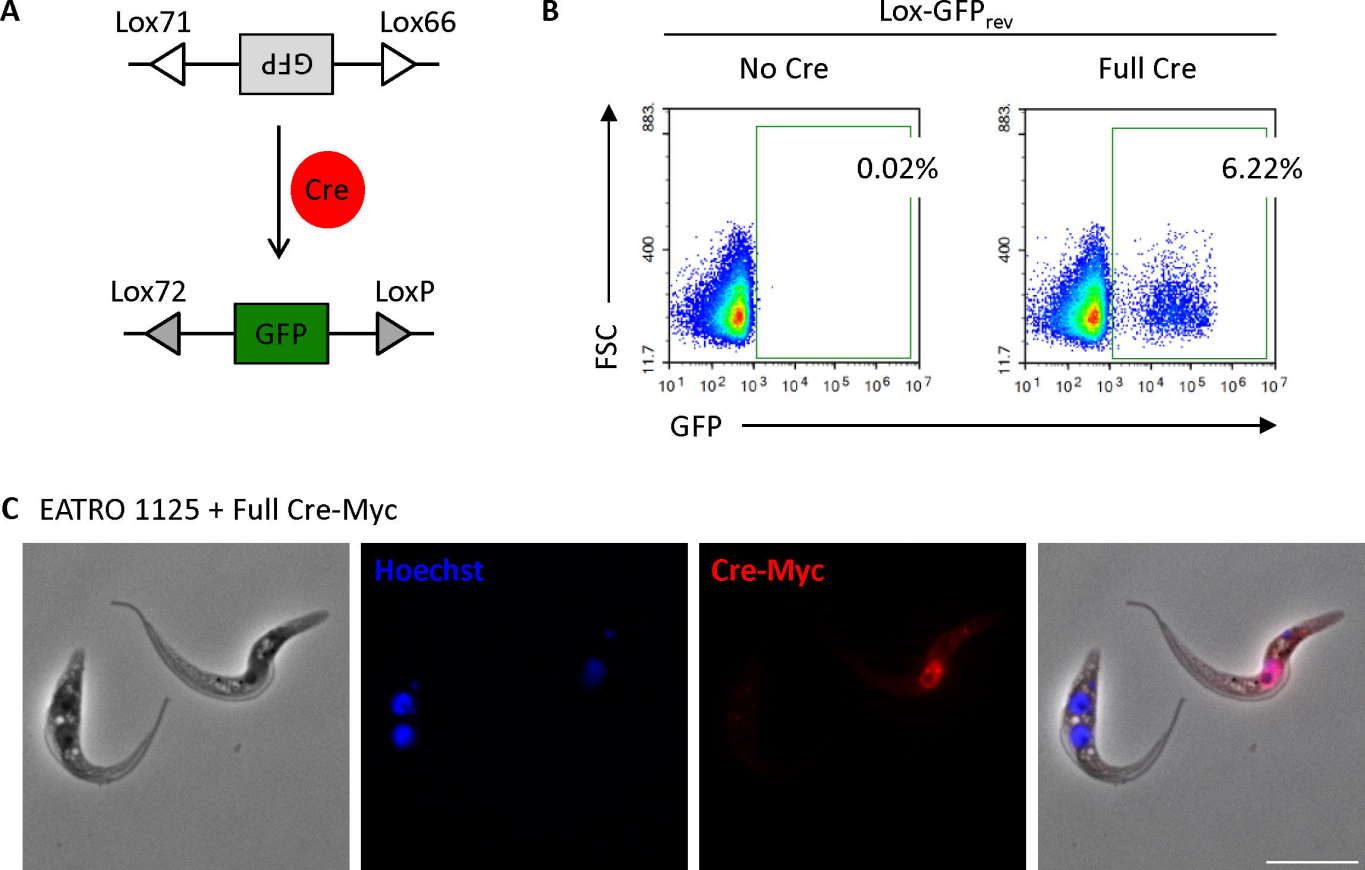
Lox-GFP_rev_ cells become GFP-positive upon Cre recombinase expression. (**A**) Schematic representation of the Lox-Cre system. (**B**) Lox-GFP_rev_ cells were analyzed by flow cytometry for GFP expression in the absence (left) or presence (right) of transient full-length Cre expression. Numbers indicate the percentage of GFP-positive cells. (**C**) Representative immunofluorescence image showing full-length Myc-tagged Cre expression in the nucleus following transient transfection. Scale bar: 10 µm.

To investigate the transient process of flagellar fusion by *T. brucei* in its tsetse fly host, we needed to establish a system to permanently label cells. To achieve this, procyclic forms of *T. brucei* EATRO 1125, a fly-transmissible strain, were stably transformed with the coding sequence for GFP in reverse orientation, flanked by Lox66 and Lox71 sites (Fig. 1A; Fig. S1A) (15, 16). These are variants of LoxP sites that allow only a single inversion event. The Lox-GFP_rev_ cassette was inserted into the genome immediately downstream of a procyclin promoter ensuring high-level transcription. Because of its reverse orientation, GFP was not synthesized and the cells were not green (Fig. 1B, left panel). We verified the functionality of Lox-GFP_rev_ by transiently transfecting these cells with full-length Cre recombinase. Flow cytometric analysis 24 h post-transfection showed that Lox-GFP_rev_ could indeed be inverted, resulting in GFP expression (Fig. 1B, right panel). The frequency of GFP-positive cells (6.22%) was in a similar range to general transient transfection rates for procyclic forms, suggesting that Cre was inverting the cassette with high efficiency. As a next step, we paired cells containing the Lox-GFP_rev_ cassette with another cell line constitutively expressing full-length Cre. These were co-cultured under conditions that allow flagellar fusion to take place. No GFP-positive cells could be detected, however (data not shown). We attributed this to the fact that Cre is tightly localized to the cell nucleus (Fig. 1C) and probably does not gain access to the flagellum for exchange during fusion.

**Fig 2:**
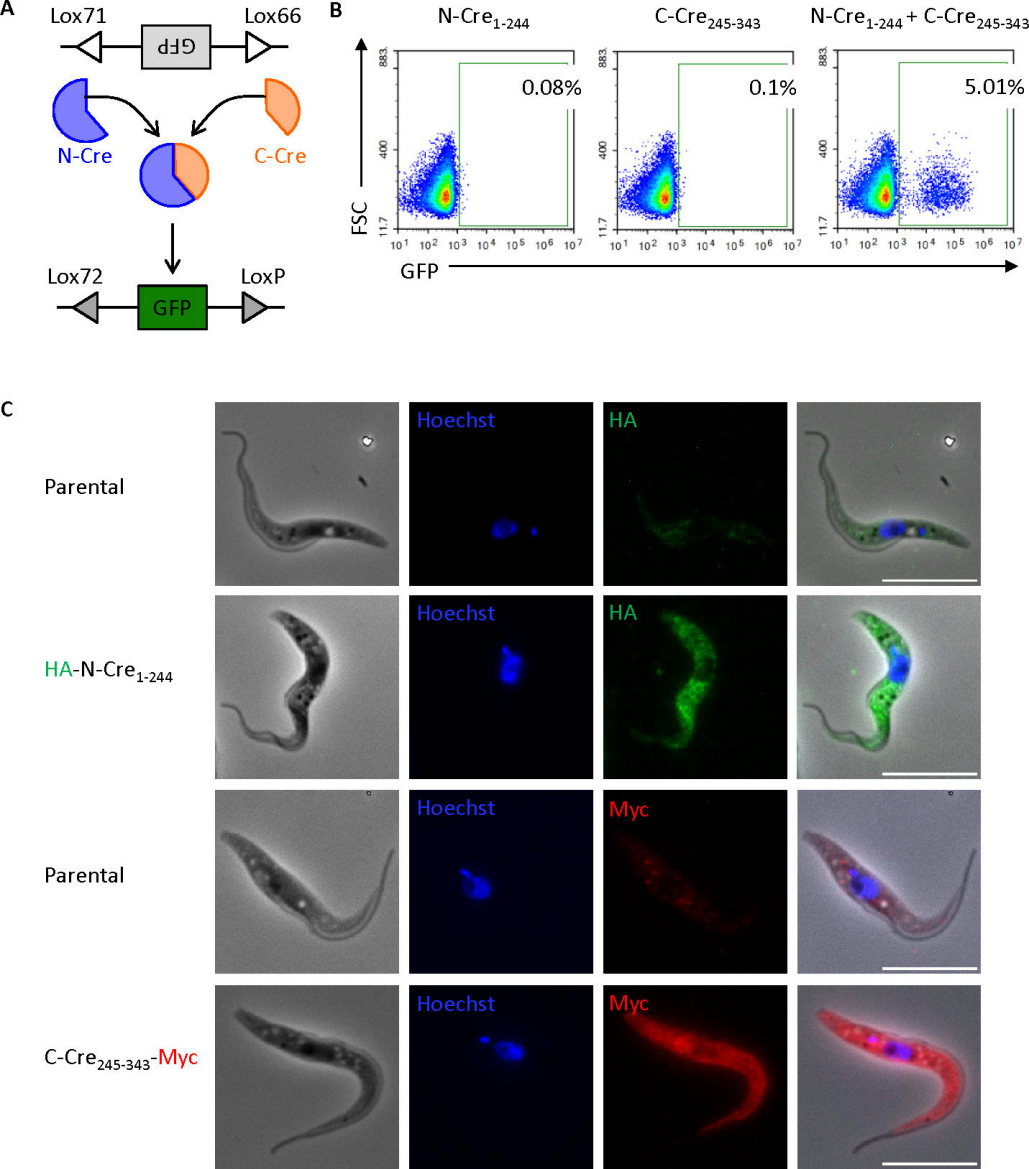
The split-Cre system is functional in trypanosomes. (**A**) Schematic representation of split-Cre used to invert GFP_rev_. The reverse GFP sequence is flanked by Lox66 and Lox71 sites. The N-terminal and C-terminal fragments of Cre assemble to form functional Cre that recognizes the Lox sites and inverts reverse GFP to its forward orientation where GFP is expressed. The inversion leads to the conversion of the Lox66 and Lox71 sites to Lox72 and LoxP sites, respectively, preventing further inversions. (**B**) Flow cytometry analysis of EATRO 1125 procyclic forms stably transformed with Lox66-GFP_rev_-Lox71 and transiently transfected with either N-Cre_1-244_, C-Cre_245-343_ or N-Cre_1-244_ plus C-Cre_245-343_. Data were acquired 24 h after transient transfection. Percentages of GFP-positive cells are indicated. (**C**) Representative fluorescence microscopy images showing HA-tagged N-Cre_1-244_ and Myc-tagged C-Cre_245-343_ expression in stably transfected EATRO 1125 cells. The parental line serves as a control for background staining. Cells were stained with Hoechst to visualize nuclei and kinetoplasts. Scale bar: 10 µm.

To circumvent this problem, we decided to split-Cre into N-terminal and C-terminal moieties (Fig. 2A), hoping that these truncated forms would gain access to the flagellum. While split-Cre has been used in many different organisms, it was not known if the separate halves would be able to pair with their partners and be functional in trypanosomes. Based on literature searches (17–20), we tested three different versions: (i) N-Cre_1-59_ and C-Cre_60-343_, (ii) N-Cre_1-196_ and C-Cre_182-343_, and (iii) N-Cre_1-244_ and C-Cre_245-343_ (Fig. S2A). To localize the proteins, we tagged each N-Cre polypeptide with an N-terminal HA tag and each C-Cre polypeptide with a C-terminal triple Myc tag. We knew that full-length Cre doubly tagged with HA and triple Myc was an active recombinase, as this was the version used in Fig. 1B. To test the functionality of the different split-Cre pairs, we transiently transfected Lox-GFP_rev_ cells with the plasmids encoding N- and C-Cre pairs, either separately or together. Twenty-four hours post-transfection, the cells were analyzed by flow cytometry for GFP expression. None of the transfections with N- or C-Cre alone resulted in GFP-positive cells, confirming that these were not functional on their own. By contrast, when we transfected the two halves simultaneously, all three pairs gave rise to green cells (Fig. 2B; Fig. S2B and C). Co-transfection with N-Cre_1-244_ and C-Cre_245-343_ led to the highest frequency of GFP-positive cells (5%). This is comparable to the value obtained with full-length Cre (Fig. 1B), despite both the N- and C-terminal halves of the protein being mainly in the cytoplasm, rather than being concentrated in the nucleus (Fig. 2C).

### Split-Cre permanently marks cells that undergo flagellar fusion *in vitro*

**Fig 3:**
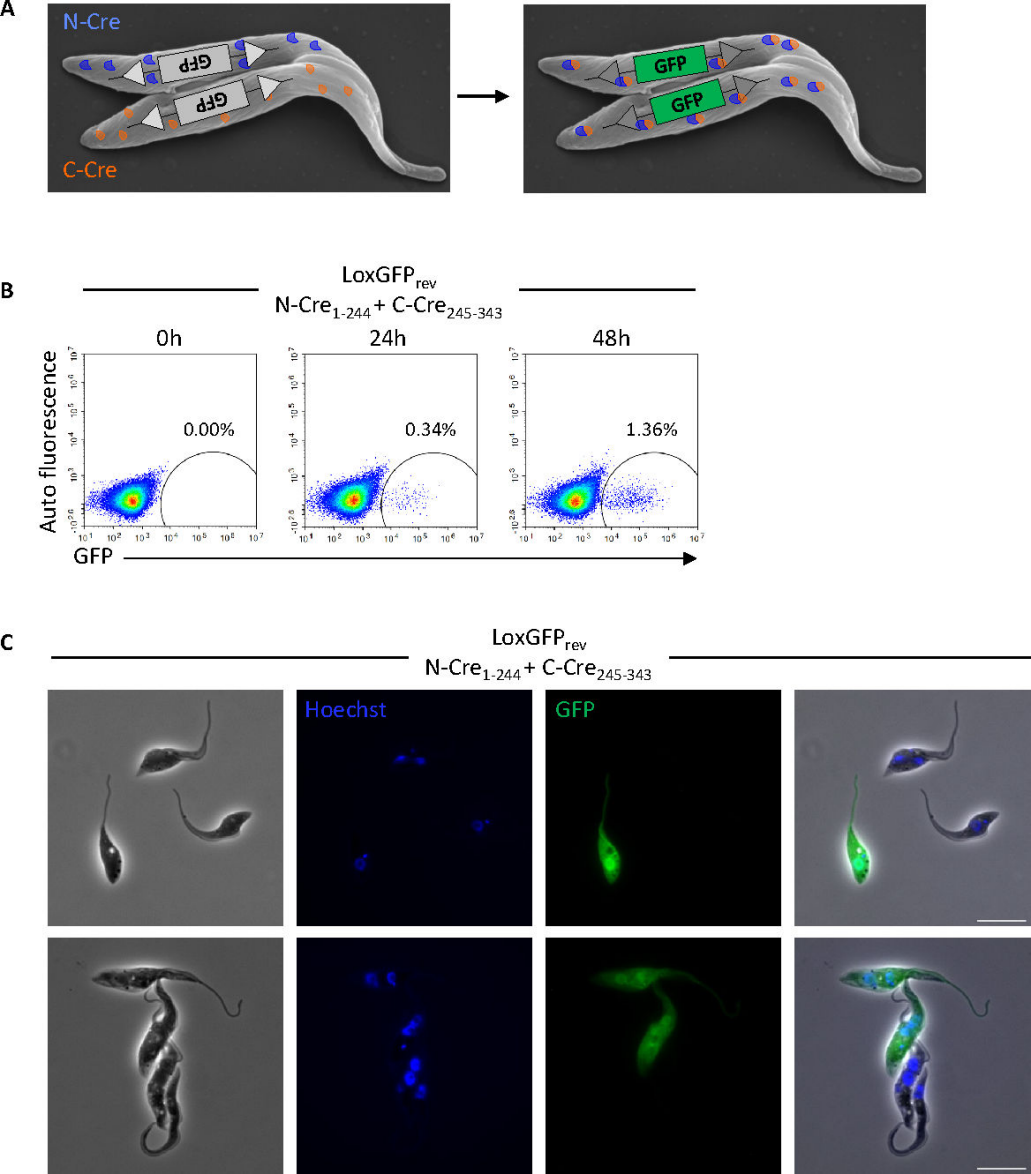
*In vitro* fusion using the split-Cre system. (**A**) Schematic view depicting N- and C-Cre exchange between two trypanosomes and inversion of GFP. Electron microscopy image from Imhof et al. (12) (**B**) Flow cytometry analysis of GFP-positive cells after 0 h, 24 h, and 48 h of co-culture of GFP_rev_ N-Cre_1-244_ and GFP_rev_ C-Cre_245-343_ cells in fusion conditions (non-heat-inactivated serum). GFP is expressed in cells that have undergone flagellar fusion. Numbers indicate percentages of GFP-positive cells. (**C**) Microscopy images of GFP_rev_ N-Cre_1-244_ and GFP_rev_ C-Cre_245-343_ cells from a 48 h fusion culture. GFP is expressed in cells that have undergone flagellar fusion. Cells were stained with Hoechst dye for visualization of nuclei and kinetoplasts. Scale bars: 10 µm.

Since transient transfection of Lox-GFP_rev_ cells with all three pairs of split-Cre produced GFP-positive cells, and the split-Cre proteins were not restricted to the nucleus, we generated stable transformants expressing individual N- or C-terminal Cre moieties in the Lox-GFP_rev_ background. To test whether Cre proteins could be exchanged between trypanosomes and produce a functional recombinase, the three split-Cre pairs were co-cultured under conditions promoting flagellar fusion (Fig. 3A). At the onset of the experiment (0 h time point), none of the cultures contained GFP-positive cells, but after 24 h, 48 h, or 72 h all three co-cultures did (Fig. 3B; Fig. S3). Once again, N-Cre_1-244_ and C-Cre_245-343_ were the most active pair, giving rise to 1.36% GFP-positive cells after 48 h (Fig. 3B); moreover, green trypanosomes could readily be detected by microscopy, including a pair with fused flagella (Fig. 3C). This frequency is comparable to what was previously observed for fusion of GFP- and DsRED-tagged cells (12). N-Cre_1-196_ and C-Cre_182-343_ were less efficient (0.27%; Fig. S3) and although GFP-positive cells could also be detected after co-culture of N-Cre_1-59_ and C-Cre_60-343_, these were very rare (Fig. S3). Imhof et al. previously reported that flagellar fusion was stimulated by one or more heat-labile factors in serum (12). In line with this, we only observed GFP-positive trypanosomes in the presence of fresh, non-heat-inactivated FBS (Fig. 3B) and not when cells were co-cultured in medium with heat-inactivated FBS (Fig. S4).

In summary, we identified a pair of split-Cre proteins, N-Cre_1-244_ and C-Cre_245-343_, that are efficiently exchanged between cells during flagellar fusion to form a functional recombinase, and can label cells permanently by inversion of the Lox-GFP_rev_ cassette.

### A selectable fusion marker to determine if both Cre halves can be transferred

**Fig 4:**
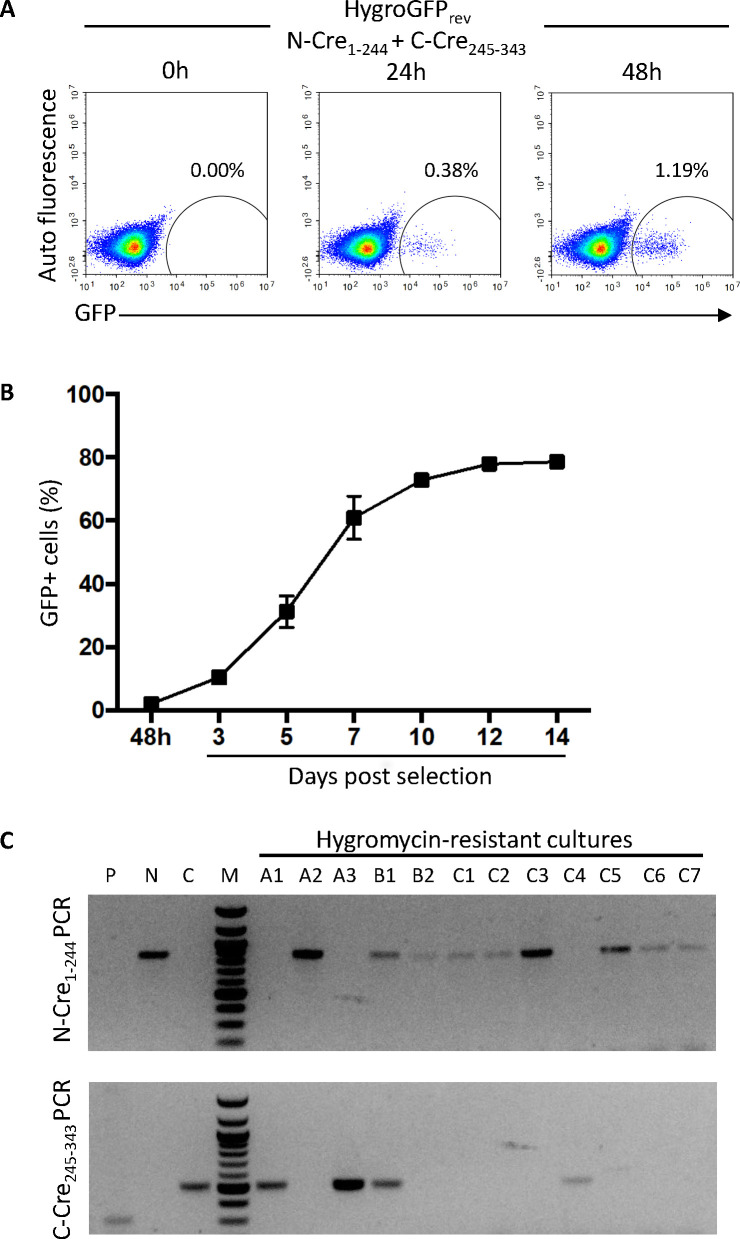
*In vitro* fusion and selection after inversion of HygroGFP_rev_ by split-Cre. (**A**) Flow cytometry analysis after 0 h, 24 h, and 48 h of HygorGFP_rev_ N-Cre_1-244_ and HygorGFP_rev_ C-Cre_245-343_ cells in fusion culture. Numbers indicate percentages of GFP-positive cells. (**B**) After 48 h in fusion culture, hygromycin was added and the frequencies of GFP-positive cells were determined by flow cytometry at the time points indicated. Means ± SD of technical triplicates are shown. (**C**) PCR analysis of N-Cre (top) and C-Cre (bottom) in the genomic DNA of GFP-positive clones resulting from a fusion experiment. P: parental wild-type cells, N: N-Cre cells without fusion, C: C-Cre cells without fusion, M: marker, A1-3, B1-2, and C1-7: hygromycin-resistant cultures isolated by limiting dilution.

Our studies so far did not allow us to determine whether both N-Cre and C-Cre were transferred by flagellar fusion. If this were the case, both cell types could act as donors and recipients, and both would express GFP after fusion. If only one half could be transferred, however, only the recipient cell would contain both Cre halves and turn green. To investigate whether both Cre halves could be exchanged, we modified the reporter cassette so that cells could be selected and cloned post-fusion. This was done by replacing GFP in the Lox-GFP_rev_ construct with a hygromycin resistance-GFP hybrid gene (Lox-HygroGFP_rev_; Fig. S1B). After generating Lox-HygroGFP_rev_/N-Cre_1-244_ and Lox-HygroGFP_rev_/C-Cre_245-343_ cell lines, we tested whether the HygroGFP protein was functional by co-culturing the two cell lines under fusion conditions. These cultures gave rise to GFP-positive cells at a similar frequency (1.19% after 48 h) to cells that inverted GFP alone (Fig. 3B and 4A). To enrich for cells that had undergone flagellar fusion, hygromycin was added to the co-cultures after 48 h and the percentage of GFP-positive cells was monitored between 3 and 14 days after the addition of the drug. The frequency of GFP-expressing cells increased to 80% of the population by day 14 (Fig. 4B).

In a second experiment, the two cell lines were co-cultured under fusion conditions for 48 h, at which point 2.2% of the cells were GFP-positive. The culture was then subjected to limiting dilution (theoretically 0.3 GFP-positive cells per well) in the presence of hygromycin. Following selection, genomic DNA was isolated from 12 wells that showed cell growth, all of which were GFP-positive (Fig. S5), and assayed by PCR for the presence of N-Cre or C-Cre (Fig. 4C). Eight samples were positive for N-Cre and three samples for C-Cre. A single sample (B1), which may not have been clonal, contained both sequences. These results demonstrate that either cell line could act as the recipient which, in turn, means that both Cre halves can move between cells during flagellar fusion.

### Monitoring flagellar fusion *in vivo*

**Fig 5:**
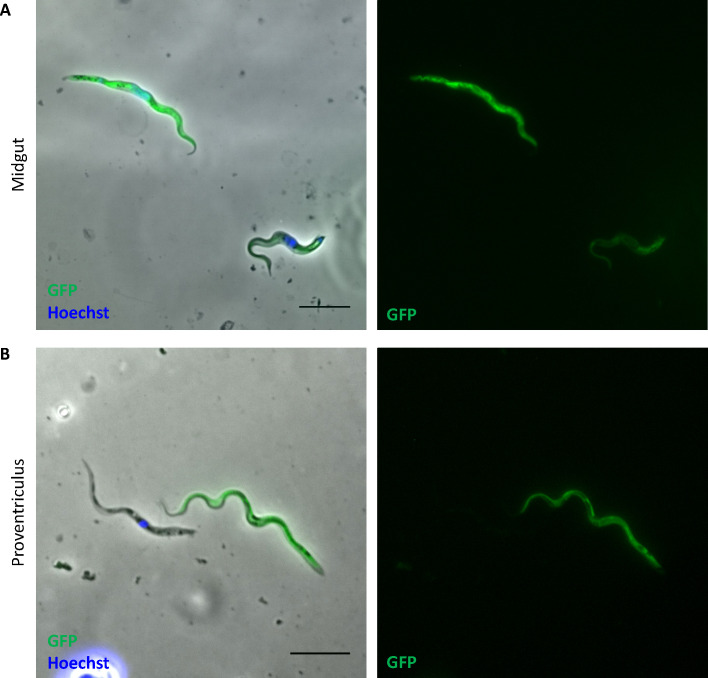
GFP-positive trypanosomes from the midgut and proventriculus of flies coinfected with Lox-GFP_rev_/N-Cre_1-244_ and Lox-GFP_rev_/C-Cre_245-343_ cells. (**A**) GFP-positive trypanosomes from a midgut sample. (**B**) GFP-positive trypanosome from a proventriculus sample. Cells were stained with Hoechst dye and analyzed by microscopy. The scale bar is 10 µm.

To detect flagellar fusion by trypanosomes in tsetse, flies were infected with a mixture of LoxGFP_rev_/N-Cre_1-244_ and LoxGFP_rev_/C-Cre_245-343_ clones at a ratio of 1:1. These were mixed immediately prior to feeding. Fusion occurs most efficiently when the parasites are exposed to non-heat inactivated serum, indicating that a labile factor plays a role. For logistical reasons, however, flies could not be fed with fresh blood. Instead, they were given tri-weekly blood meals of cold-stored defibrinated blood. From day 28 to day 34 post-infection, the midguts, proventriculi, and salivary glands of infected flies were analyzed for GFP-positive trypanosomes by fluorescence microscopy. Out of 89 flies with a midgut infection, we found two samples with green trypanosomes (Fig. 5A). Similarly, out of 79 infected proventriculi, one contained GFP-positive cells (Fig. 5B; Table S2). The fact that these cells were not in pairs with a fused flagellum indicates that they had either separated again or were descendants of cells that had fused previously. These observations demonstrate that fusion can already take place in the midgut, which is consistent with it occurring in procyclic forms in culture. We did not detect GFP-positive trypanosomes in the salivary glands, but infections of the glands overall were very low. In control experiments, flies were infected with either LoxGFP_rev_/N-Cre_1-244_ or LoxGFP_rev_/C-Cre_245-343_ cells alone. GFP-positive trypanosomes were never detected in these flies, confirming that GFP_rev_ does not flip spontaneously to the forward orientation.

**Fig 6:**
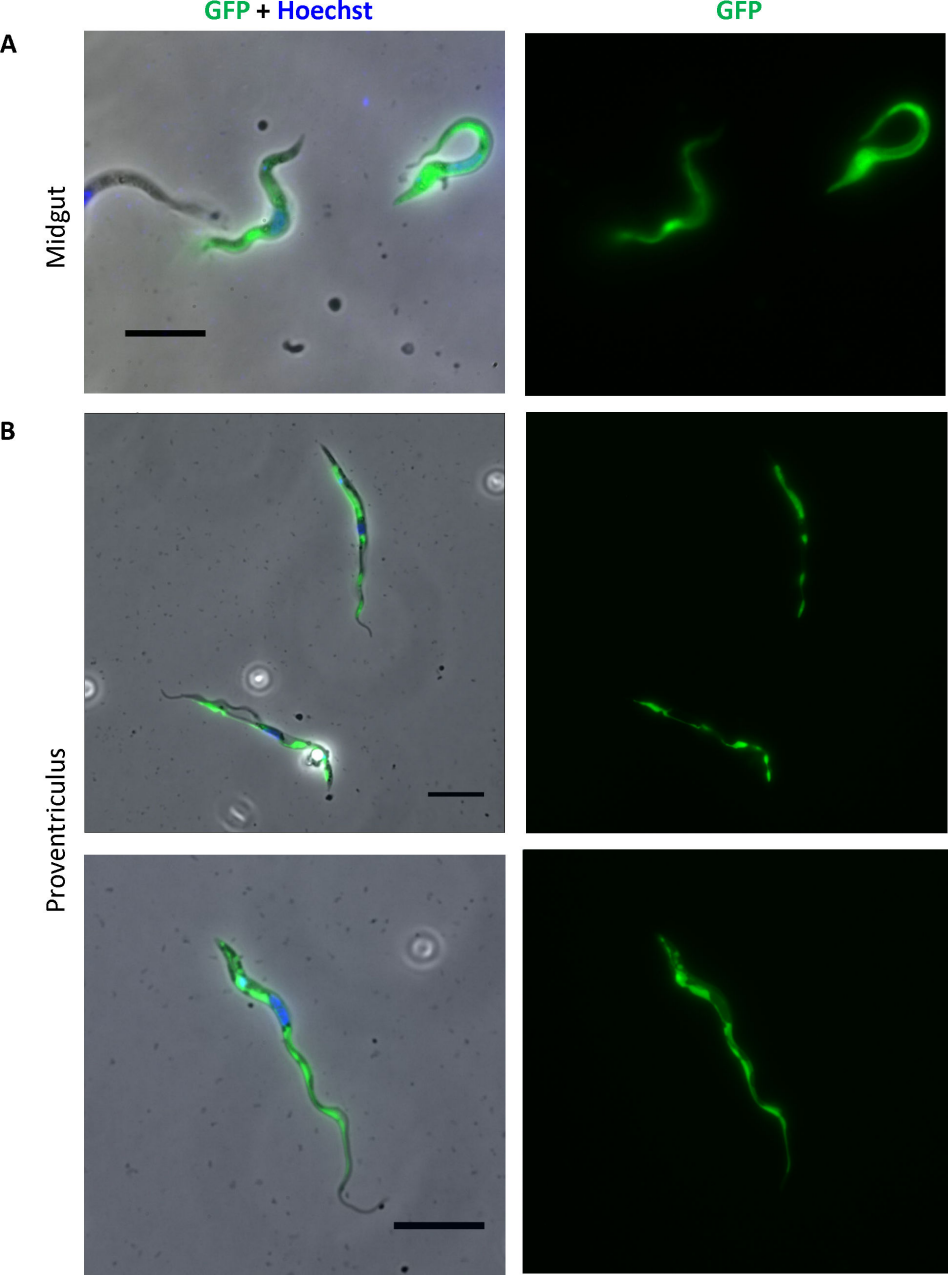
GFP-positive trypanosomes from the midgut and proventriculus of flies coinfected with Lox-HygroGFP_rev_/N-Cre_1-244_ and Lox-HygroGFP_rev_/C-Cre_245-343_ cells. (**A**) GFP-positive trypanosomes from a midgut sample. (**B**) GFP-positive trypanosomes from a proventriculus sample. Cells were stained with Hoechst dye and analyzed by microscopy. The scale bar is 10 µm.

When tsetse flies were co-infected with Lox-HygroGFP_rev_/N-Cre_1-244_ and Lox-HygroGFP_rev_/C-Cre_245-343_, green trypanosomes were detected in two proventriculi and two pools of midguts on day 28 post-infection (Fig. 6). In contrast to GFP alone, however, the HygroGFP hybrid protein was not homogeneously distributed within the cytoplasm. The most likely explanation for this difference is that the hygromycin resistance domain is the main determinant of the protein’s localization within the parasites.

Many tsetse flies do not harbor trypanosomes after taking an infectious blood meal. The use of reduced glutathione is well established for improving midgut infection rates to almost 100% (21), increasing the likelihood of detecting events that occur at low frequencies. Therefore, in a third experiment, midgut infection rates were enhanced by the inclusion of 10 mM reduced glutathione in an infective bloodmeal containing Lox-HygroGFP_rev_/N-Cre_1-244_ and Lox-HygroGFP_rev_/C-Cre_245-343_ cells. Of the 143 flies with infections of both the midgut and proventriculus, no green cells were found in the midgut, but six flies had green trypanosomes in the proventriculus (Table S2).

Taken together, these data show that flagellar fusion occurs *in vivo* but, at least under the conditions used, these events are rare. It is worth noting, however, that in any case where a binary system is used, only half of the cells that have undergone flagellar fusion will be detected. Fusions of N-Cre with N-Cre cells or C-Cre with C-Cre cells will not reconstitute full Cre and will not be able to flip the floxed cassette to its forward orientation. In some cases, the fluorescence intensity in some cells was fainter than in others (Fig. 5A). This might reflect the transfer of cytoplasmic contents from a green cell that had fused previously, and inverted Lox-GFP_rev_ or Lox-HygroGFP_rev_, to a recipient cell. If the donor and recipient cells contained the same half of Cre, the transferred GFP would be degraded or diluted over time.

## DISCUSSION

We have established a split-Cre system to permanently mark trypanosomes that have undergone flagellar fusion *in vitro* or *in vivo* and have demonstrated that flagellar fusion happens *in vivo* in the tsetse fly. Of the three pairs of split-Cre that were tested all were active, either when expressed in the same cell, or transferred between cells by flagellar fusion. Cells that assembled a functional Cre recombinase could invert a floxed cassette, leading to high-level constitutive expression of GFP. We showed for the most efficient pair, N-Cre_1-244_ and C-Cre_245-343_, that both can be transferred to recipient cells, meaning that any fusion between cells expressing these two proteins could invert the reporter gene and activate GFP expression in both partners. This pair was also tested in co-infections of tsetse flies, resulting in the detection of GFP-positive cells in midguts and proventriculi in three independent experiments. This is consistent with our findings that procyclic forms fuse their flagella and exchange contents in culture. Based on their morphology, many of the green cells observed *in vivo* were mesocyclic forms. These follow procyclic forms in the life cycle *in vivo* but are not normally seen *in vitro*. It is not clear whether these cells were the primary cells that had fused or if they had differentiated from procyclic forms that had fused previously.

The mechanism and biological role of flagellar fusion remain enigmatic, but there are several reasons why it might occur (previously summarized in reference 12). Direct transfer between cells could prevent proteins and metabolites from being diluted or from being detected by the host immune system. It might also enable parasites to selectively obtain or deliver missing nutrients. This phenomenon has been described for cross-feeding bacteria, which exchange metabolites via tight junctions or nanotubes. The fact that the argonaute protein was shown to move between cells (12) raises the possibility that RNAs associated with it can also be transferred. Both RNA-binding proteins and non-coding RNAs can induce differentiation in trypanosomes (22–24).

Flagellar fusion undoubtedly occurs *in vivo*, although it may not be a common event under laboratory conditions for tsetse fly infections. Careful inspection of transmission electron micrographs in the literature, performed for other reasons (25, 26), identified several examples of *T. brucei* flagellar fusion events in different tissues in the tsetse fly (Fig. S6). One cross-section from the tsetse midgut ectoperitrophic space showed two examples, one with the flagellar membrane surrounding two axonemes and paraflagellar rods and another with three axonemes and paraflagellar rods (Fig. S6A). Another example, from a different study and laboratory, showed two axonemes and paraflagellar rods surrounded by a single membrane in a trypanosome sample from the proventriculus (Fig. S6B). In our study, we did not observe pairs of cells with fused flagella among the trypanosomes we extracted from the different tsetse tissues, but this may have been for technical reasons. On the one hand, our experiments focused on identifying green cells. If cells fused on a random basis, only half of the fusion events would bring together N-Cre and C-Cre. When this does occur, there is likely to be a lag between fusion, inversion of the floxed cassette by the two halves of Cre, and the expression of GFP. As we have shown previously, fusion is stimulated by one or more heat-labile factors in serum (12). It is possible that these factors are depleted from the defibrinated blood used to feed the flies, decreasing the frequency of fusion *in vivo*. In general, laboratories maintaining tsetse have moved to membrane feeding of stored defibrinated blood, rather than using animals. By contrast, flies in the wild feed every few days on fresh blood, where the stimulatory factors might be present in higher concentrations. It is also possible that individual tsetse influenced fusion since all flies received the same blood, yet only a few harbored green trypanosomes. This heterogeneity might reflect their genetic background and/or the endosymbiont population.

The split-Cre system we describe here, particularly the version with Lox-HygroGFP_rev_, which enables the selection of low-frequency events, could be used for additional experiments and in other contexts. For example, rare GFP-positive cells that had undergone flagellar fusion in the fly could be selected *in vitro* for further studies. Unfortunately, the challenges associated with culturing trypanosomes that were isolated from the fly were beyond the scope of this study. In addition, the Lox-HygroGFP_rev_ system could be employed to investigate whether flagellar fusion by *T. brucei* occurs in the bloodstream or various tissues of the mammalian host. It could also be applied to other parasites, for example, to determine whether intracellular parasites release proteins into their host cell. In this case, the floxed reporter gene would be in the host cell genome and the host cell would become GFP-positive. It could also be applied to trans-well assays to determine whether vesicles shed by a parasite fuse with host cells.

It is sometimes argued that if an event is infrequent, it is unlikely to be biologically relevant, but there are many examples of rare events where this is clearly not the case. Trypanosomes experience a massive bottleneck in their progression from the midgut to the salivary glands, with the result that a single trypanosome can be the progenitor of >99% of the population in the glands (27). This is biologically relevant in two ways. First, most of the parasites that initially infected a fly will not complete their life cycle and be transmitted to a new mammalian host. Second, the parasite(s) reaching the glands first will dominate the population that is transmitted by that fly. There are also many examples of rare events in human biology that have major consequences. Mutation of a single amino acid in the epidermal growth factor receptor can determine whether tumor cells are sensitive or refractory to an anti-cancer drug (28). Single nucleotide changes can confer both advantages and disadvantages depending on the context. These include sickle cell mutations in human hemoglobin genes, which make heterozygotes more resistant to malaria, while homozygotes experience debilitating symptoms (29). Single nucleotide mutations in the human apolipoprotein LI gene can confer resistance to infection by *T. b. gambiense*, but make their carriers more prone to kidney disease (30). Last but not least, the existence of every one of us comes down to an exceedingly rare event—the one sperm in approximately 10^8^ that fused with and fertilized an egg.

## MATERIALS AND METHODS

### Cell culture

Procyclic forms of *T. brucei brucei* EATRO 1125, initially derived from AnTat 1.1 bloodstream forms (31), were used for this study. Procyclic forms were cultured at 27°C in SDM79 (32) containing 10% heat-inactivated FBS and 20 mM glycerol. Parasites were maintained at cell densities between 2 × 10^6^ and 2 × 10^7^ cells mL^−1^.

### Generation of constructs

A list of oligonucleotides is provided in Table S1. Plasmid sequences, determined by Microsynth AG (Balgach, Switzerland), will be provided on request. To generate the pG-Lox71-GFP_rev_-Lox66-ΔLII-Neo construct (Lox-GFP_rev_), a construct with the coding region of GFP flanked by Lox66 and Lox71 in the forward orientation was first generated. EcoRI-Lox66-HindIII oligonucleotides (Table S1; Fig. S1, (15, 16)) were annealed and cloned between the HindIII/PinAI sites of pG-GFP-ΔLII-Neo (12), destroying both sites in the process, and resulting in pG-Lox66-GFP-ΔLII-Neo. Next BamHI-Lox71-EcoRI oligonucleotides (Table S1; Fig. S1A) were annealed and cloned into the BamHI site of pG-Lox66-GFP-ΔLII-Neo, destroying the 5′ Bam HI site but leaving the 3′ BamHI site intact. The correct orientation of the Lox71 oligonucleotides was verified by sequencing. The resulting construct, pG-Lox66-GFP_fwd_-Lox71-ΔLII-Neo was then digested with EcoRI, to excise GPF, and re-ligated yielding 50% pG-Lox66-GFP_fwd_-Lox71-ΔLII-Neo and 50% pG-Lox71-GFP_rev_-Lox66-ΔLII-Neo (Fig. S1). The reverse orientation of GFP was verified by PCR.

The HygroGFP fusion gene was generated by fusion PCR. The hygromycin gene from pLEW100v5_HYG (https://tryps.rockefeller.edu/trypsru2_plasmids.html) was amplified using oligonucleotides HindIII-Hygro-Fwd and Hygro(GFPtail)-Rev; GFP was amplified from pG-Lox66-GFP_fwd_-Lox71-ΔLII-Neo with oligonucleotides eGFP(Hygro-tail)-Fwd and eGFP-BamHI-Rev. The two PCR products were combined in a subsequent PCR reaction using HindIII-Hygro-Fwd and eGFP-BamHI-Rev primers and the resulting product was cloned between the HindIII and BamHI sites of pG-Lox71-GFP_rev_-Lox66-ΔLII-Neo replacing GFP_rev_.

A construct containing full-length Cre with an N-terminal HA tag and a C-terminal triple Myc tag was generated by amplifying full-length Cre from pLEW100-Cre (33) with a forward primer containing the HA tag (HindIII-HA-Cre Fwd) and a reverse primer without the stop codon (BglII-Cre-noStop Rev). The PCR product was digested with Hind III and Bgl II and cloned into pSAK21:C-term3xMyc (a gift from S. Käser and A. Schneider, University of Bern) to give rise to pSAK21-HA-Cre-3xMyc.

HA-tagged N-terminal Split-Cre constructs were generated by amplifying HA-Cre from pSAK21-HA-Cre-3xMyc with the HA-Cre forward primer and a reverse primer inside Cre, introducing a stop codon. The PCR products were cloned into the BamHI site of pG-mcs-Blast, a derivative of pG-mcs-ΔLII (34), to generate pG-HA-N59-Cre-Blast, pG-HA-N196-Cre-Blast and pG-HA-N244-Cre-Blast. Myc-tagged C-terminal split-Cre constructs were generated by amplifying Cre-Myc from pSAK21-HA-Cre-3xMyc with forward primers within the Cre gene, introducing a start codon, and a reverse primer annealing to the triple Myc tag. PCR products that contained the full triple Myc tag were gel-purified and cloned into pG-mcs-Blast to generate pG-C60-Cre-3xMyc-Blast, pG-C182-Cre-3xMyc-Blast and pG-C245-Cre-3xMyc-Blast.

### Transfections

Stable transfections were performed by electroporation with 10 µg linearized plasmids as previously described (35) in Tb-BSF buffer (36). All plasmids used for stable transfection were derived from pGAPRONE; transcription is driven by a procyclin promoter (37). Integration occurs upstream of the procyclin locus on either chromosome 6 or 10. Plasmids were linearized with SpeI and clones were selected with 15 µg mL^−1^ G418 or 10 µg mL^−1^ blasticidin, as appropriate.

Transient transfections were performed by electroporating 4 × 10^7^ cells with 10 µg supercoiled plasmid in Tb-BSF buffer (36) using an Amaxa Nucleofector 2b, program X-014 (Lonza, Switzerland). After electroporation cells were cultured for 24 h or 48 h followed by analysis by flow cytometry or microscopy.

### Flow cytometry and immunofluorescence

For analysis of GFP expression, 50–100 µL of cell culture was diluted with phosphate-buffered saline (PBS) to a total volume of 500 µL to obtain 1–4 × 10^6^ cells mL^−1^. Data were acquired using a NovoCyte Flow Cytometer (Bucher Biotec) and analyzed using NovoCyte Novoexpress software v1.6.2.

Rat anti-Myc antibody clone 9E10 was purchased from Thermofisher Scientific and mouse anti-HA clone 3F10 from Sigma-Aldrich. For immunofluorescence, cells were harvested by centrifugation, washed once with PBS, settled on coverslips, fixed in 4% paraformaldehyde in PBS, and permeabilized with Triton X-100 (0.2% in PBS). After blocking in PBS/3% BSA for 1 h, cells were incubated with primary antibody (1:250 for anti-HA, 1:50 for anti-Myc) overnight at 4°C, washed three times in PBS and subsequently incubated with secondary antibody (1:2,000, AlexaFluor488-donkey-anti-rat, ThermoFisher or Cy3-goat-anti-mouse, Invitrogen) for 1 h at room temperature. Cells were then washed three times in PBS and stained with Hoechst 33342 prior to embedding in Mowiol (Sigma-Aldrich). Fluorescent images were captured using a Leica DM5500 B microscope and images were superimposed using ImageJ 1.54f.

### Flagellar fusion assays and selection of hygromycin-resistant cells

Non-heat-inactivated FBS was purchased from Sigma-Aldrich. Prior to use in fusion assays, aliquots were stored at −20°C, then thawed at 27°C. For flagellar fusion experiments, pairs of clones (1.5 × 10^7^ cells each) were mixed and centrifuged for 8 min at 2,500 rpm (1,300 × *g*) in a Heraeus Megafuge 1.0R. The supernatant was removed, and the cells were resuspended in 9 mL SDM79 without FBS. 1 mL of non-heat-inactivated FBS was added and cells were cultured at 27°C. Cells were monitored by flow cytometry or microscopy for GFP expression after 24 h, 48 h, or 72 h. Selection of hygromycin-resistant cells in bulk cultures: following co-culture of Lox-HygroGFP_rev_/N-Cre_1-244_ and Lox-HygroGFP_rev_/C-Cre_245-343_ for 48 h under fusion conditions, hygromycin (25 µg mL^−1^) was added to the medium. Cells were passaged by dilution to 3 × 10^6^ mL^−1^ at intervals of 2–3 days and analyzed by flow cytometry to determine the frequency of GFP-positive cells.

Isolation of hygromycin-resistant clones: after 48 h co-culture of Lox-HygroGFP_rev_/N-Cre_1-244_ and Lox-HygroGFP_rev_/C-Cre_245-343_, the percentage of GFP-positive cells was determined by flow cytometry. Limiting dilution was performed by distributing cells (theoretically 0.3 GFP-positive cells per well) in 96-well plates. These were cultured together with 10^5^ feeder cells (wild-type procyclic forms) in 100 µL SDM79 supplemented with 10% heat-inactivated FBS, 20 mM glycerol, and 20% conditioned medium. After 20 h, 100 µL SDM supplemented with 10% heat-inactivated FBS, 20 mM glycerol, and 50 µg mL^−1^ hygromycin was added to each well. After 15–20 days of hygromycin selection, 12 positive wells were picked and passaged three times (1:10 dilution in the presence of hygromycin). Genomic DNA was isolated using standard procedures and PCR amplified using diagnostic primers for N-Cre_1-244_ (SPU and N-Cre-244-BglII Rev) and C-Cre_245-343_ (SPU and Myc-BamHI Rev; see Table S1).

### Tsetse fly infections

*Glossina morsitans morsitans* pupae were obtained from the Department of Entomology, Slovak Academy of Science, Bratislava, Slovakia or the Institute of Tropical Medicine, Antwerp, Belgium (Table S2). Teneral flies were offered two infectious blood meals, 24 h and 48 h after eclosion, by membrane feeding with 2.5  ×  10^6^ procyclic forms mL^−1^ resuspended together with washed defibrinated horse red blood cells (TCS Biosciences, UK) or sheep red blood cells (E & O Laboratories Ltd, UK). Flies were maintained at 24°C and fed three times per week with defibrinated blood. From days 28 to 34 post-infection, midguts, proventriculi, and salivary glands were dissected and monitored for the presence of trypanosomes. Positive midgut samples were collected in Falcon tubes by filtering through 100 µm cell strainers (BD Falcon), washed twice with PBS, stained with Hoechst dye, and embedded in Mowiol. Positive proventriculus samples were immediately embedded in Mowiol containing Hoechst dye. Samples were analyzed for GFP-positive trypanosomes using a Leica DM5500 B microscope; phase contrast and fluorescent images were superimposed using FiJi v1.52p.

## SUPPLEMENTAL MATERIAL

Supplemental Material mbio.03375-24-s0001.pdf Figures S1-S6 and Tables S1 and S2.

ASM does not own the copyrights to Supplemental Material that may be linked to, or accessed through, an article. The authors have granted ASM a non-exclusive, world-wide license to publish the Supplemental Material files. Please contact the corresponding author directly for reuse.
