# Supplementary material for: Split-Cre-mediated GFP expression as a permanent marker for flagellar fusion of Trypanosoma brucei in its tsetse fly host
Source: mBio. 2024 Dec 17;16(2):e03375-24. doi: 10.1128/mbio.03375-24 (PMC11796343; doi:10.1128/mbio.03375-24)
Supplement: Supplemental Material — Figures S1-S6 and Tables S1 and S2. [file mbio.03375-24-s0001.pdf]

Supplementary Figure 1

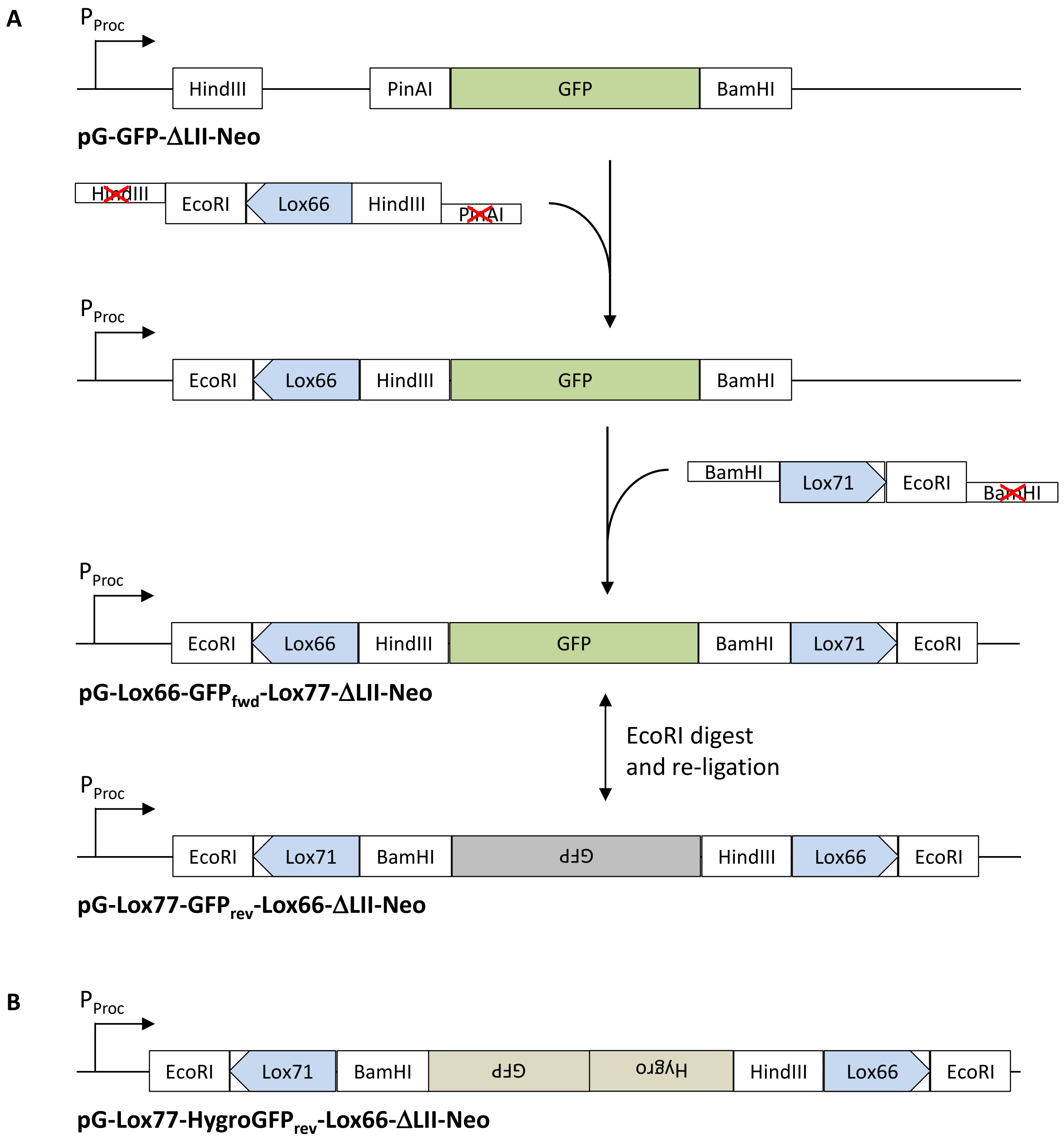

Supplementary Figure 1

Cloning strategy. (A) To generate the pG-Lox71-GFP_{rev}-Lox66-ΔLII-Neo construct, a construct with GFP flanked by Lox66 and Lox71 in the forward orientation was first generated. EcoRI-Lox66-HindIII oligonucleotides were annealed and cloned between the HindIII/PinAI sites of pG-GFP-ΔLII-Neo, destroying the HindIII and PinAI sites used for cloning, resulting in pG-Lox66-GFP-ΔLII-Neo. Next, BamHI-Lox71-EcoRI oligonucleotides were annealed and cloned into the BamHI site of pG-Lox66-GFP-ΔLII-Neo, destroying the 5' BamHI site, but leaving the 3' BamHI site intact. pG-Lox66-GFP-Lox71-ΔLII-Neo was then digested with EcoRI and re-ligated. This resulted in 50% pG-Lox66-GFP_{fwd}-Lox71-ΔLII-Neo and 50% pG-Lox71-GFP_{rev}-Lox66-ΔLII-Neo. The orientation of GFP in individual plasmids was determined by PCR. (B) To generate pG-Lox71-HygroGFPrev-Lox66-ΔLII-Neo, GFP in the pG-Lox71-GFPrev-Lox66-ΔLII-Neo plasmid was replaced with HygroGFP.

Supplementary Figure 2

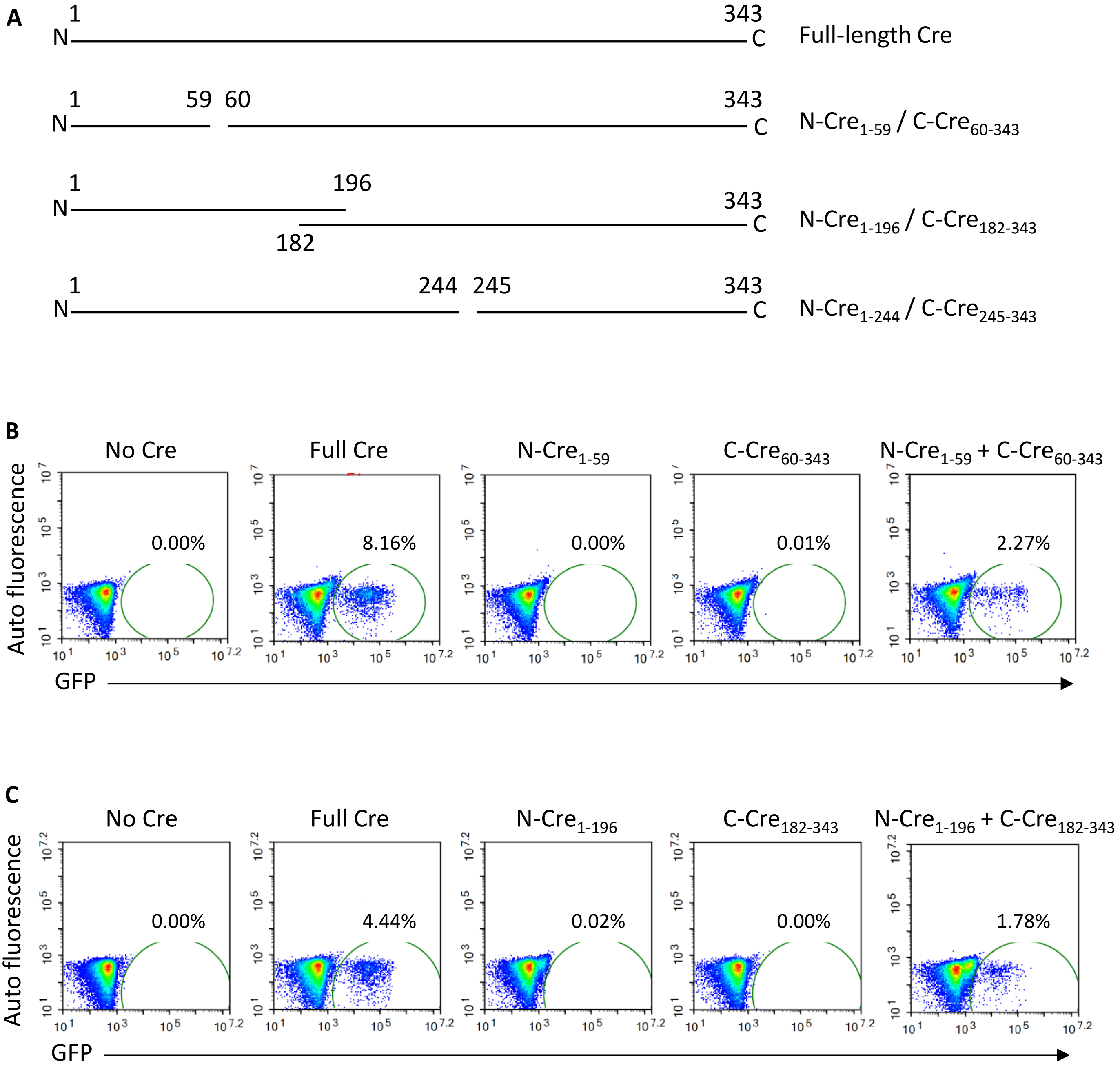

Supplementary Figure 2

Testing different versions of split-Cre. (A) Schematic view of the different pairs of split-Cre that were tested. (B) and (C) Flow cytometry analysis of EATRO 1125 cells stably transfected with Lox66-GFPrev-Lox71 and transiently transfected with either full-length Cre, N-Cre$_{1-59}$, C-Cre$_{60-343}$ or N-Cre$_{1-59}$ plus C-Cre$_{60-343}$ (B) or full-length Cre, N-Cre$_{1-196}$, C-Cre$_{182-343}$ or N-Cre$_{1-196}$ plus C-Cre$_{182-343}$ (C). Data were acquired 24h after transient transfection. Percentages of GFP-positive cells are indicated.

Supplementary Figure 3

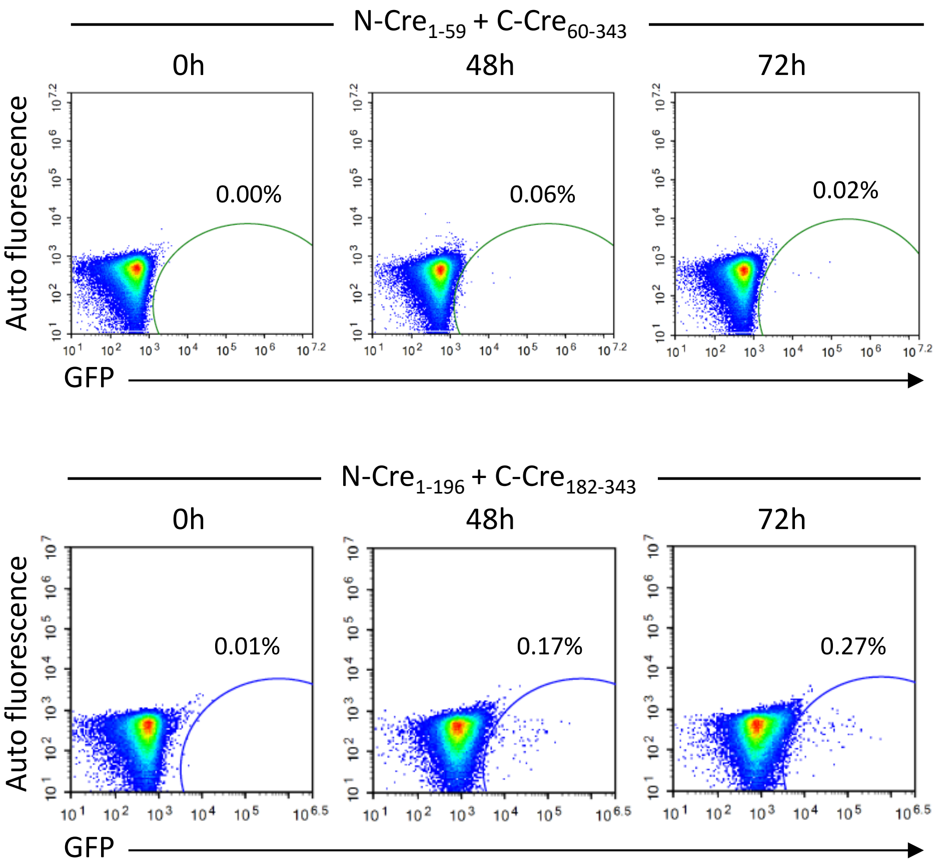

Supplementary Figure 3

Flagellar fusion experiments for other split-Cre cell lines. Flow cytometry analysis for GFP-positive cells from LoxGFP$_{rev}$ N-Cre$_{1-59}$ and LoxGFP$_{rev}$ C-Cre$_{60-343}$ cells (top) or LoxGFP$_{rev}$ N-Cre$_{1-196}$ and LoxGFP$_{rev}$ C-Cre$_{182-343}$ cells (bottom) after 0h, 48h and 72h in fusion culture. Numbers indicate percentages of GFP-positive cells.

Supplementary Figure 4

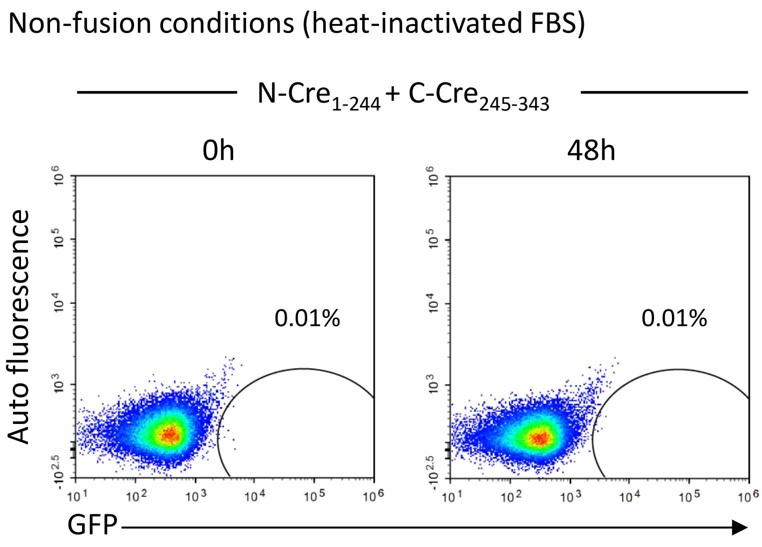

Supplementary Figure 4

Background flagellar fusion in cultures with heat-inactivated serum. Flow cytometry analysis of HygroGFP$_{rev}$ N-Cre$_{1\text{-}244}$ and HygroGFP$_{rev}$ C-Cre$_{245\text{-}343}$ cells co-cultured for 0h and 48h in medium containing heat-inactivated FBS (non-fusion condition).

Supplementary Figure 5

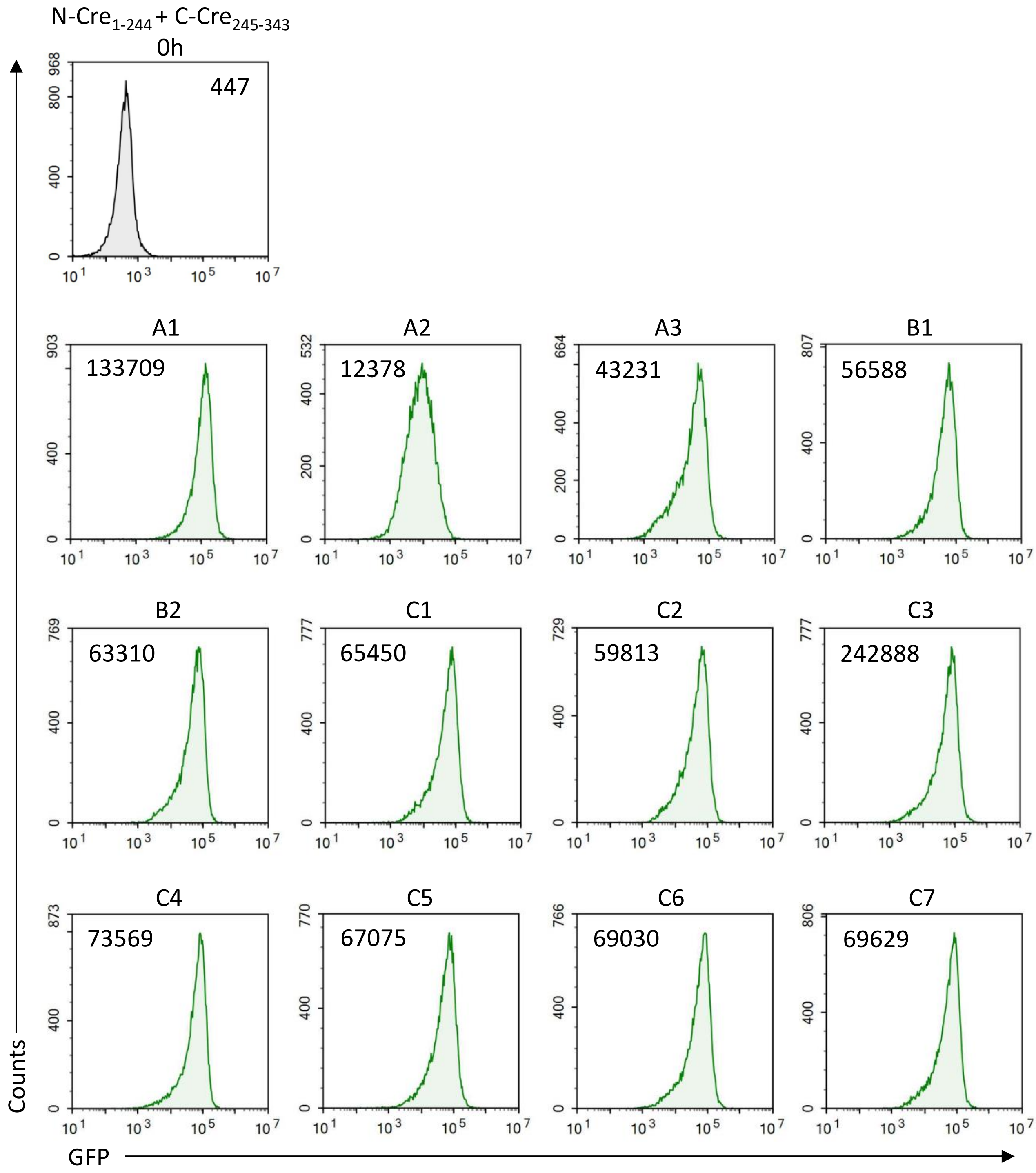

Supplementary Figure 5

Flow cytometry analysis for GFP expression of hygromycin-resistant cultures from Figure 4C.

Numbers in plots indicate mean fluorescent intensities (MFI).

Supplementary Figure 6

A

B

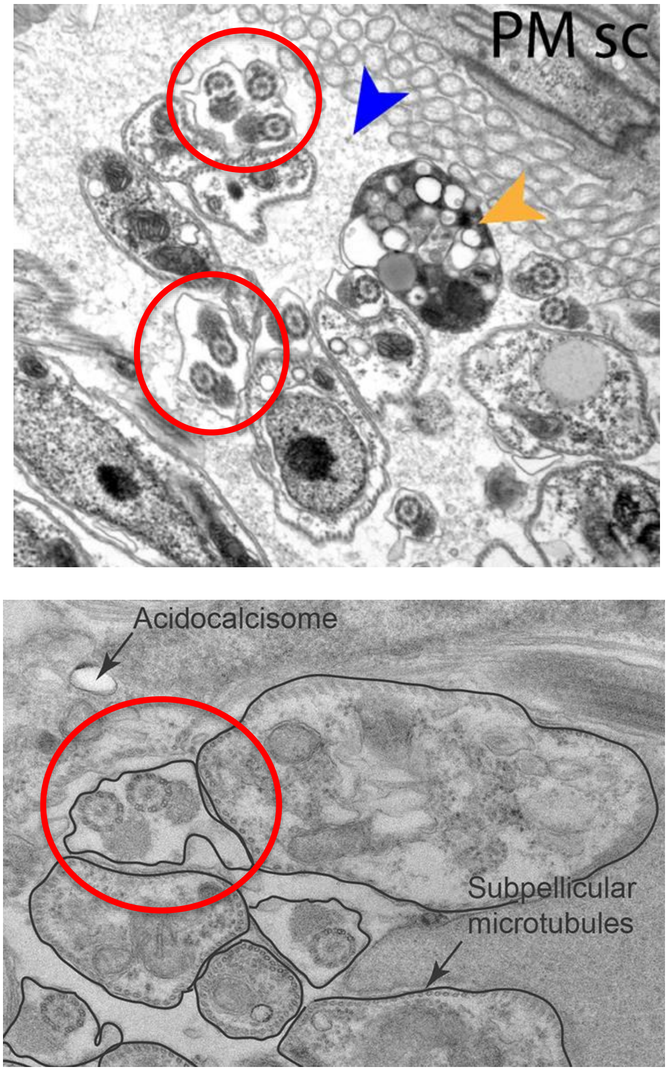

Supplementary Figure 6

Examples of flagellar fusion *in vivo* found in previously published electron micrographs.

(A) From Vigneron et al., 2019 (25). Coloured arrowheads are from the original figure. (B) From Bertiaux et al., 2020 (26). Fused flagella are encircled in red. Permission to reproduce panel A according to CC BY 4.0 licence. Panel B reproduced / adapted with permission from the Journal of Cell Science (jcs.biologists.org) DOI:10.1242/jcs.248989.

Supplementary Table 1

Primer sequences used in this study.

Primer name	Primer sequence
Lox66-oligo-Fwd	AGCTGAATTCATAACTTCGTATAGCATACATTATACGAACGGTAAAGCTT
Lox66-oligo-Rev	CCGGAAGCTTTACCGTTCGTATAATGTATGCTATACGAAGTTATGAATTC
Lox77-oligo-Fwd	GATCCTATAACTTCGTATAATGTATGCTATACGAACGGTAGAATTC
Lox77-oligo-Rev	GATCGAATTCTACCGTTCGTATAGCATACATTATACGAAGTTATA
HindIII-Hygro-Fwd	ccaaagcttATGAAAAAGCCTGAACTC
Hygro(GFPtail)-Rev	cgcccttgctcaccatTTCCTTTGCCCTCGGAC
eGFP(Hygro-tail)-Fwd	gtccgagggcaaaggaaATGGTGAGCAAGGGCG
eGFP-BamHI-Rev	gttggatccTTACTTGTACAGCTCGTCC
HindIII-HA-Cre Fwd	CCaagcttATGTACCCTTACGACGTACCTGACTACGCTggtgctTCCAATTTACTGACCG
BglII-Cre-noStop Rev	AGGagatctATGGTTATCGCCATCTTC
N-Term-59-Cre Rev	agtGGATCCtcaATTCAACTTGCACCATG
C-Term-60-Cre Fwd	gcaAAGCTTatgAACCGGAAATGGTTTCC
C-Term-Myc-BglII Rev	tggAGATCTTCACAGCAGATCCTCC
N-Cre-196-BglII Rev	tggAGATCTtcaATGGATTAACATTCTCCC
C-Cre-182-HindIII Fwd	gcaAAGCTTatgGTTAAAGATATCTCACGTAC
Myc-BamHI Rev	agtGGATCCTCACAGCAGATCCTCC
N-Cre-244-BglII Rev	tggAGATCTtcaTTTTCTGACCCGGCA
C-Cre-245-HindIII Fwd	gcaAAGCTTatgAATGGTGTTGCCGCG
SPU	GCTGCACGCGCCTTCGAGTT

Supplementary Table 2

Summary of fly experiments. MG: Midgut, PV: Proventriculus, SG: Salivary glands.

**Experiment**	**1**	**2**	**3**
**Floxed cassette**	eGFP	HygroGFP	HygroGFP
**L-glutathione***	no	no	yes
**Source of pupae**	Bratislava	Antwerp	Antwerp
**Site of experiment**	Bern	Bern	Antwerp
**Bloodmeal (species)**	Horse	Horse	Sheep
**Number MG +**	89	27	145
**Number PV +**	79	25	143
**Number SG +**	13	2	0
**GFP positive samples**	3	4	6

*10mM reduced glutathione was included in the infective blood meal.
